# Supplementary material for: A sustainable lithium bromide-water absorption cooling system using automobile engine waste heat: Theoretical study
Source: PLoS One. 2025 May 23;20(5):e0324800. doi: 10.1371/journal.pone.0324800 (PMC12101701; doi:10.1371/journal.pone.0324800)
Supplement: Attachment — Submitted filename: Response to Reviewers.docx [file pone.0324800.s002.docx]

**Responses to dear reviewers**

Dear Reviewers, I would like to express my thanks and gratitude to you for evaluating the research paper submitted by me, as I benefited from your wonderful and insightful information that contributes to increasing the strength of the research paper's outputs.

**Manuscript: A sustainable lithium bromide-water absorption cooling system using automobile engine waste heat: Theoretical study**

Reply to the first reviewer

1. The study is entirely theoretical. The authors should either provide experimental data to validate the model, or compare their results with published experimental studies to demonstrate the models reliability.

Thank you for your comment. We acknowledge the importance of experimental data for validating theoretical models. However, we have compared our results with published experimental studies, specifically with Florides et al., and Patel et al., as shown in Table 4 and 5. This comparison demonstrates that our theoretical results align well with the experimental data from their study, validating the reliability and accuracy of our model. By comparing with Florides et al. [43] and Patel et al. [44]we have shown that the proposed model is consistent with experimental observations, ensuring its applicability and robustness.

2. The manuscript references multiple previous experimental studies; however, it does not quantitatively compare its results with real-world data (e.g., COP values, temperature ranges, emission reductions). Given the large body of research on LiBr-H₂O systems, this omission weakens the credibility of the findings.

Thank you for your feedback. We have performed a quantitative comparison with the study by Ammar et al., as shown in Table 6. Our results showed good agreement with theirs, demonstrating the reliability of our model. We compared COP values, temperature ranges, and emission reductions, and the comparison indicated consistent trends, validating the robustness of our approach.

3. The novelty of the paper is not clear. The equations and modeling approach follow conventional thermodynamic principles without introducing significant new computational methodologies. The analysed temperature range is typical for an absorption system, and the system itself also looks conventional. Therefore, the authors are asked to enhance the explanation of why this paper should be published?

Thank you for your thoughtful feedback. We acknowledge that the study primarily follows established thermodynamic principles. However, we would like to emphasize that the novelty of our paper lies in the specific application and optimization of conventional thermodynamic models rather than introducing entirely new computational methodologies.

a. Secondary Heat Exchanger: A significant contribution of our work is the integration of a secondary heat exchanger into the absorption system, which results in a 5.4% improvement in generator load. This simple but effective optimization has the potential to improve the performance of existing absorption systems, making it an important practical contribution.
b. Real-World Applications: While the temperature range analyzed is typical for absorption systems, our study provides insights into the behavior of the system under specific, real-world conditions. This is particularly useful for the design of more efficient systems in modern applications where precise operating conditions are crucial.
c. Environmental Impact: The comparison of emission reductions between gasoline and diesel engines in absorption systems provides new insights into how different fuels affect system performance and emissions. This comparison can help guide future developments in environmentally friendly absorption systems.

In conclusion, the novelty of our work lies not in developing entirely new methodologies but in applying existing principles in a way that offers new insights, optimizations, and environmental benefits. We believe that these contributions justify the publication of this paper, as they have practical implications for improving the efficiency and sustainability of absorption systems.

4. The manuscript does not include an error analysis. The authors should assess how sensitive the results are to input parameters, estimate potential uncertainties, and compare their findings with experimental data to ensure their accuracy.

Thank you for the valuable comment.

A detailed sensitivity analysis was included in Section 4.5 of the manuscript, where the effects of key input parameters—such as generator temperature, first heat exchanger effectiveness, and weak solution concentration—on the system's performance were investigated. The outcomes of this analysis are clearly illustrated in Figures 11, 13, and 14, which demonstrate how these inputs influence the COP and generator load, highlighting the importance of carefully controlling these parameters to achieve higher operational efficiency. In addition, the accuracy of the developed numerical model was validated by comparing the calculated COP values with results reported by Florides et al., as shown in Figure 12. The comparison revealed good agreement within acceptable relative deviations. The reliability of the model was further supported by comparison with previously published theoretical results (Table 3) and experimental data from the literature (Tables 4 and 5), which collectively confirm the validity and robustness of the proposed thermodynamic model.

Reply to the second reviewer

1. The temperature should be clearly mentioned in the abstract and manuscript. Because only single temperature has been mentioned for generator, absorber, condenser and evaporator. However, the variation of temperatures is shown in result.

I would like to thank the reviewer for their valuable comment. In this study, a single temperature was mentioned for the generator, absorber, condenser, and evaporator to simplify the basic model and ensure clarity. However, temperature variations were considered in the analysis presented in the results, where dynamic temperature changes based on different operational conditions were shown. These clarifications were made to provide a clear understanding of how temperature variations affect the system's performance, while a fixed temperature was used in the abstract to present the core concept clearly without delving into detailed specifics.

2. Define the objective of the study clearly. Rewrite your abstract.

I would like to sincerely thank the reviewer for their valuable comments. Based on the feedback provided, the text has been revised to clarify the objective of the study in a more precise and clear manner. The objective of this study is to analyze and evaluate an absorption cooling system (ACS) in which automotive engine waste heat is utilized as the energy source. The study also investigates the effect of integrating an additional heat exchanger between the existing heat exchanger and the generator, which utilizes engine coolant heat. Furthermore, the impact of temperature variations in the main components and changes in solution concentration on system efficiency is analyzed under various operating conditions. Finally, the system is evaluated in terms of its environmental benefits, including emission reductions and the potential for sustainability through decreased fuel consumption. The abstract and the objective of the study have been revised and clearly presented at the end of the introduction section.

3. Mention the boundary condition and limitation of the study in methodology.

Thank you for this valuable comment. Based on your observation, the assumptions (boundary conditions) have been gathered in the theoretical analysis section of the manuscript, where they are clearly presented in 7 points. Additionally, the limitations and operating conditions are detailed in Table 1. Furthermore, sensitivity analysis of the model has been added in the results section, where the impact of input variations on the model's outputs and the system's sensitivity are discussed.

4. Modify the figure 2. Axis titles are missing. Also cite the manuscript from which this study was validated. It is not an ethical way to write “another research.

Thank you for this valuable comment. Based on your observation, the figure with the missing axis titles has been removed, while keeping Table 3,4 and 5 to compare the validity of the results of our mathematical model with both a theoretical model and an experimental model, as shown in the tables. Additionally, we will fully adhere to ethical standards and ensure proper citation of sources in the manuscript

5. Show the standard deviation for bar chat graphs.

Thank you for your valuable comment. Figure 2 has been removed from the manuscript, while the values of the variance or discrepancy are provided in Tables 3, 4 and 5. Readers can refer to these tables for the variance associated with the data.

6. From figure 5-11, there are several sub-figures available. But these figures are not mentioned in captions. So, mention all the figures in figure caption.

Thank you for your valuable comment. Based on your suggestion, I will revise the figure captions for figures 5-11 to include all the sub-figures. Each sub-figure will be clearly mentioned in the captions to ensure clarity and proper reference within the manuscript.

7. Check the heading numbers of the manuscript.

Thank you for your valuable comment. The heading numbers in the manuscript have been checked and corrected to ensure consistency and proper sequence.

8. In figure 11, SOx and NOx reduction is not clearly visible. So, modify the figure11 in such a way that all data should clearly visible in manuscript.

We appreciate your constructive feedback. Based on your suggestion, Figure 11 has been removed and replaced with a more accurate graph that shows the reduction in emissions over the course of the year see figure (10). This modification ensures better clarity of the emission reduction data in the manuscript.

9. Highlight the major findings of the study in the bullet point in conclusion section and also mention that how this study will help the society.

Thank you for pointing that out. The requested changes have been made, where the major conclusions have been summarized and how this study can benefit society has been clearly outlined. All points have been addressed in detail in the revised manuscript.

10. Check the grammatical errors in the manuscript.

Thank you for your valuable comment. The requested changes have been made, and the manuscript has been revised for linguistic and grammatical accuracy to ensure clarity and precision in the text.
